# Head and Neck Paragangliomas: Overview of Institutional Experience

**DOI:** 10.3390/cancers16081523

**Published:** 2024-04-16

**Authors:** Swar N. Vimawala, Alex Z. Graboyes, Bonita Bennett, Maria Bonanni, Aleena Abbasi, Tanaya Oliphant, Michelle Alonso-Basanta, Christopher Rassekh, Debbie Cohen, Jason A. Brant, Yonghong Huan

**Affiliations:** 1 Division of Otolaryngology-Head and Neck Surgery, Cooper University Health Care, Camden, NJ 08103, USA; vimawala-swar@cooperhealth.edu; 2 Department of Otorhinolaryngology-Head and Neck Surgery, Perelman School of Medicine, University of Pennsylvania, Philadelphia, PA 19104, USA; alex.graboyes@pennmedicine.upenn.edu (A.Z.G.); tanaya.oliphant@pennmedicine.upenn.edu (T.O.); christopher.rassekh@pennmedicine.upenn.edu (C.R.); 3 Renal, Electrolyte and Hypertension Division, Perelman School of Medicine, University of Pennsylvania, Philadelphia, PA 19104, USA; bonita.bennett@pennmedicine.upenn.edu (B.B.); maria.bonanni@pennmedicine.upenn.edu (M.B.); debbie.cohen@pennmedicine.upenn.edu (D.C.); 4 Department of Radiation Oncology, Perelman School of Medicine, University of Pennsylvania, Philadelphia, PA 19104, USA; aleena.abbasi@pennmedicine.upenn.edu (A.A.);; 5 Corporal Michael J. Crescenz VA Medical Center, Philadelphia, PA 19104, USA

**Keywords:** paraganglioma, head and neck paraganglioma, carotid body, jugular paraganglioma, vagal paraganglioma, tympanic paraganglioma, head and neck tumors, succinate dehydrogenase mutations

## Abstract

**Simple Summary:**

Head and neck paragangliomas (HNPGLs) are rare and have high rates of genetic mutations. SDHx mutations currently account for the vast majority of mutations identified in HNPGLs, and SDHB and SDHD are the two most common types. Surgery remains the definitive treatment, while radiation therapy is being increasingly used as an adjuvant or primary treatment for surgically challenging or inoperable cases. Our study provided clinical and outcome data on a large cohort of patients with HNPGLs and the results can help provide guidance on improving the care and outcomes of patients with HNPGL.

**Abstract:**

Head and neck paragangliomas (HNPGLs) are rare and have high rates of genetic mutations. We conducted a retrospective review of 187 patients with 296 PGLs diagnosed between 1974 and 2023. The mean age of diagnosis was 48.8 years (range 10 to 82) with 69.0% female and 26.5% patients with multiple PGLs. Among 119 patients undergoing genetic testing, 70 (58.8%) patients had mutations, with SDHB (30) and SDHD (26) being the most common. The rates of metastasis and recurrence were higher among patients with SDHB mutations or SDHD mutations associated with multiple PGLs. Metabolic evaluation showed elevated plasma dopamine levels were the most common derangements in HNPGL. MRI and CT were the most common anatomic imaging modalities and DOTATATE was the most common functional scan used in this cohort. Most patients (81.5%) received surgery as the primary definitive treatment, while 22.5% patients received radiation treatment, mostly as an adjuvant therapy or for surgically challenging or inoperable cases. Systemic treatment was rarely used in our cohort. Our single-center experience highlights the need for referral for genetic testing and metabolic evaluation and for a team-based approach to improve the clinical outcomes of patients with HNPGLs.

## 1. Introduction

Paragangliomas of head and neck and skull base (HNPGLs) are rare, mostly slow-growing, and hypervascular tumors arising from neural crest-derived cell clusters located along the jugular foramen, middle ear, carotid bifurcation and vagal nerve, among other locations [1,2,3]. HNPGLs are also commonly known as glomus tumors, though this is a misnomer as they are not true glomus tumors. They are known to account for 0.6% of all head-and-neck-related cancers [1,2]. The incidence of these tumors is low—approximately 1–8 cases per million people [4,5].

The presentation of symptoms of HNPGLs is extremely variable and depends upon anatomic location. Within the neck region, carotid body PGLs (CBPs) typically arise at the carotid bifurcation, while vagal PGLs (VPs) typically arise from the inferior vagal ganglion. Patients with CBPs or VPs typically present with neck mass, cough, hoarseness, or dysphagia [6,7]. Tympanic PGLs (TPs) arise from the tympanic plexus of Arnold’s and Jacobson’s nerve along the cochlear promontory [8]. Jugular PGLs (TPs) arise from the jugular bulb [9]. As both TPs and JPs occur in the middle ear, patients typically present with otalgia, pulsatile tinnitus, hearing loss, and in the latter’s case, potentially lower cranial nerve deficits due to proximity to glossopharyngeal and other lower cranial nerves. While there have been up to 20 locations reported in the literature for HNPGL, CBPs, VPs, JPs, and TPs are the most common [10]. Up to 60% of HNPGLs are CBPs [1].

Up to 95% of HNPGLs are nonsecretory, with it being even rarer for TPs [2,11]. However, HNPGLs are often associated with succinate dehydrogenase (SDHx) genetic variation [12]. SDHx germline mutations have been identified in up to 40% of HNPGLs [13,14,15]. A systematic review with meta-analysis by Brito et al. demonstrated that genetic mutations are present in roughly 11–13% of those who presented with a sporadic pheochromocytoma or PGLs (PPGLs) [16]. Those with genetic mutations are more prone to developing multiple tumors [10,15,17,18].

In recent years, we have developed a better understanding of HNPGLs and their clinical behavior. The management of these tumors is therefore evolving; although surgery remains the primary form of treatment, nonsurgical treatments are being utilized more frequently. The purpose of this study was to review our institutional experience in the management of patients with HNPGLs and the changing trends in how we treat patients, especially those with genetic mutations.

## 2. Methods

We performed a retrospective analysis of a cohort of patients who received care at our institution for a diagnosis of HNPGL made between 1974 and 2023. Within this patient cohort, information was collected on all PGLs diagnosed in all body locations alongside the clinical information of each patient including family history, and genetic, metabolic, imaging, and treatment data. Demographic data such as age of diagnosis, gender, and race/ethnicity were also collected. Family history included family members with a diagnosis of a PGL or a known related mutation. Genetic data included genetic testing and identified mutations. Metabolic data included all available results on plasma or urine catecholamines and metanephrines. The metabolic data were categorized as within normal limits (WNL), greater than one but less than three times the upper limit of normal (1–3 ULN), or greater than three times of the ULN (>3 ULN). The metabolic results were reported in aggregate using the highest category for individual and overall tests. Imaging studies included diagnostic and surveillance imaging encompassing CT, MRI, angiogram, and nuclear medicine imaging such as DOTATATE, MIBG, FDG-PET, or FDPA-PET. Treatment data included definitive and salvage therapy including surgery, radiation, radionuclide and medical therapy.

## 3. Data Analysis

This study primarily reports descriptive statistics on this patient cohort with a primary focus on genetic, metabolic, imaging, and treatment data. Student’s *t*-test was performed to analyze differences in the age of diagnosis between patients with and without genetic testing and patients with and without mutations. Additionally, Chi-squared analysis was performed to compare patients with single or multiple tumors and mutation status. All statistical calculations were carried out using IBM SPSS Statistics (Version 28).

## 4. Results

### 4.1. Study Cohort

Table 1 lists the demographic information of the study cohort. The cohort had 187 patients, with the majority being female (N = 129, 69.0%) and Caucasian (N = 140, 74.9%). The mean age of initial diagnosis was 48.8 (±17.4) years for the entire cohort, while the mean ages of initial diagnosis were much lower, at 44.1 (±17.8), for the group with genetic testing and 34.6 (±13.7) years for the group with identified mutations.

There were a total of 296 PGLs identified in the entire cohort with an average of 1.6 (±1.4) PGLs per patient. Table 2 summarizes the demographic information based on tumor types. The distribution of PGLs in this cohort was carotid body PGL (CBP, N = 108), jugular PGL (JPs, N = 64), tympanic PGL (TP, N = 34), vagal PGL (VP, N = 23), other HNPGL (N = 17), and non-HNPGL (N = 50). Of the 50 non-HNPGL, 9 were located in the chest, 11 adrenal pheochromocytoma, and 30 were extra-adrenal abdominal paragangliomas. Of these 30 extra-adrenal abdominal paragangliomas, 18 were para-aortic. The group of non-HNPGL had the youngest mean age of diagnosis at 40.8 ± 15.5 years, while the group of TP had the oldest mean age of diagnosis at 55.4 ± 13.7 years. While the majority (69.0%) of the cohort was female, TP was almost exclusively diagnosed in female patients (N = 33, 97.1%).

### 4.2. Genetic Data

Among the entire cohort of 187 patients, 119 (63.6%) patients underwent genetic testing specifically for PGLs and 70 (59%) patients had positive results. Female patients were less likely to have genetic testing (OR 0.50, 95%CI 0.25–0.99, *p* < 0.05). Even when female patients underwent genetic testing, they had lower odds of having positive results (OR 0.29, 95% CI 0.12–0.66, *p* < 0.01). There were no significant differences in the rates of genetic testing and positive results of genetic mutations among different race/ethnicity groups.

Patients with a positive family history of PGLs had significantly higher odds of undergoing genetic testing (OR 24.6, 95%CI 3.3–185.0, *p* < 0.01) and higher odds of positive results of genetic mutations (OR 38.2, 95%CI 5.0–292.2, *p* < 0.01). Additionally, patients with genetic testing had a significantly higher number of tumors compared to patients without genetic testing, 1.9 ± 1.6 vs. 1.0 ± 0.2 (*p* < 0.01), respectively. Moreover, patients with genetic mutations had a higher mean number of PGLs per patient than patients without genetic mutations, 2.4 ± 1.9 vs. 1.2 ± 0.6 (*p* < 0.01), respectively.

Among the group of 33 patients (17.6%) with a known family history of PGL, all patients underwent genetic testing (N = 33, 100.0%) and an extremely high rate of positive identification of genetic mutations (N = 32, 97.0%). Among the group of 154 (82.4%) patients without a known family history, only 56.5% (87/154) patients underwent genetic testing but close to half of those (39/87, 44.8%) had positive identification of a mutation.

The group of non-HNPGL had the highest rate (52.0%) of association with family history, followed by the groups of other HNPGL (41.2%), VP (34.8%), CBP (27.8%), JP (14.1%), and TP (2.9%). While almost the entire group of non-HNPGL (N = 49, 98.0%) underwent genetic testing, the rates of genetic testing were lower for groups of HNPGLs, especially low for TPs at only 23.5%. The rate of positive identification of genetic mutation was the highest for the group of other HNPGLs (93.3%) followed by the groups of non-HNPGL (89.8%), VP (81.0%), CBP (69.0%), JP (58.7%), and TP (25.0%).

Table 3 and Table 4 summarize the results for genetic mutation. The most identified genetic mutations in the cohort were in succinate dehydrogenases (SDHx), including SDHB (N = 30, 42.9%), SDHD (N = 26, 37.1%), SDHC (N = 7, 10.0%), SDHA (N = 2, 2.9%). SDHB and SDHD were by far the most identified genetic mutations in our cohort, as shown in Table 4. The patients with SDHD mutations had much higher odds of having multiple PLGs compared to patients with SDHB mutations (OR 6.29, 95% CI 1.74–22.71). In addition, patients with multiple PGLs were more likely to have a positive identification of genetic mutation compared to patients with single PGL (OR 6.45, 95CI 2.64–15.73, *p* < 0.01). While the rates of metastasis and recurrence associated with SDHB mutations seemed comparable for patients with both single and multiple PGLs, the rates of metastasis and recurrence associated with SDHD were significantly higher in patients with multiple PGLs compared to patients with single PGL.

### 4.3. Metabolic Data

Plasma metabolic testing was available in 154 patients and urine metabolic testing was available in 84 patients. Table 5 and Table 6 provide the plasma and urine metabolic results by types of PGLs. While the plasma epinephrine and metanephrine levels were mostly normal in the cohort across the different types of PGLs, a significant portion of other HNPGLs and non-HNPGLs were associated with >3 ULN norepinephrine levels (20.0% vs. 15.4%, respectively) and >3 ULN normetanephrine levels (33.3% vs. 38.5%, respectively). Moreover, dopamine levels were elevated at 3 > ULN in a significant portion of all types of PGLs, including 27.7% of CBPs, 36.5% of JPs, 18.2% of TPs, 15.8% of VPs, 33.3% of other HNPGLs, and 42.3% of non-HNPGLs.

Similar patterns were observed from urine metabolic data, though the overall rates of elevated test levels were much lower compared to plasma results. Urine epinephrine and metanephrine levels were mostly normal but a significant portion of other HNPGLs and non-HNPGLs had >3 ULN norepinephrine levels (10.0% vs. 20.0%, respectively) and >3 ULN normetanephrine levels (18.2% vs. 18.8%, respectively). Again, dopamine levels were at >3 ULN in 4.9% of CBPs, 3.8% of JPs, 9.1% of other HNPGLs, and 20.0% of non-HNPGLs.

### 4.4. Diagnostic Imaging

Of the 296 primary tumors in this cohort, 258 had accessible imaging results related to the diagnosis and workup (Table 7A,B). Anatomic imaging defined as MRI, CT, US, or angiogram was performed alone or in combination for 237 primary tumors (91.9%). Functional imaging such as DOTATATE, FDG-PET, FDFA-PET and MIBG was performed alone or in combination for 125 primary tumors (48.4%). Ultrasound (30/30, 100%) and angiography (21/32, 65.6%) were used almost exclusively in the diagnostic workup of CBPs. DOTATATE is the most commonly used functional imaging study in this cohort (96/125, 76.8%). Functional imaging such as MIBG was used most frequently in the setting of a non-HNPGL (12/26, 46.2%).

### 4.5. Treatment

Table 8 summarizes the treatment information for the cohort. Treatment data were available in 294 PGLs (294/296, 99.3%). Local therapy defined as surgery and/or radiation therapy was performed in 227 primary PGLs. Surgery alone was performed in 125 PGLs and together with embolization in 58 PGLs. ERBT was performed in 22 PGLs, while SRS was performed in 19 PGLs. JPs were frequently treated with radiation therapy (23/54, 42.6%) compared to other PGLs, which were primarily treated with surgery. Medical treatment (N = 5) or radionuclide with MIBG (N = 3) was performed for eight PGLs. A total of 60 primary tumors are under active surveillance, which is approximately 20.3% of all primary tumors (Table 8A,B). Additionally, two patients were lost to follow-up and their treatment data were unavailable.

## 5. Discussion

The current cohort includes 187 patients with 296 primary PGLs with slightly more than twice as many females as males with an average age at diagnosis of 48.8 ± 17.4 years. Erickson et al. similarly reported a cohort of 236 patients with 297 paragangliomas evaluated at Mayo Clinic over 1978–1998 [1]. Their study also showed a female preponderance of 60% and a similar average age at diagnosis of 47 ± 16 years of age [1]. A similar cohort was published by Papaspyrou et al. including 175 patients with 224 HNPGLs evaluated at their institution between 1989 and 2010 [19]. They reported a similar ratio of females to males of 66.3% to 33.7% alongside an average age of 42 years at diagnosis [19]. Additionally, Rijken et al. in 2019 published a single-center experience including 147 patients with 289 HNPGLs over a 60-year period and 68% of their cohort were female and had an average age of 45.3 years at diagnosis [20]. Overall, there seems to be a female preponderance (60–68%) for PGLs with an average age of presentation at 42–56 years [1,19,20,21,22,23,24].

The distribution of HNPGLs in our cohort included 108 (43.7%) CBP, 64 (25.9%) JP, 34 (13.8%) TP, 23 (9.3%) VP, 18 (7.3%) other HNPGL. These results were similar to those of previous studies that showed a higher prevalence of CBP (30–57%) and JP (23–39%) compared to TP (6–14%) or VP (11–20%) [1,19,20].

Guidelines released on PPGLs by the Working Group on Endocrine Hypertension of the European Society of Hypertension recommend genetic testing to be performed on all patients with PGL due to the high rates of germline mutations found in this population [25]. Multiple genes have been implicated, with hereditary forms of HNPGL including SDHB, SDHD, SDHC, SDHA, SDHAF2, VHL, TMEM127, RET, NF1, and MAX [12]. Among the 119 patients who underwent genetic testing for PGLs in our cohort, 70 (58.5%) patients had a mutation identified. These mutations include SDHB (N = 30, 42.9%), SDHD (N = 26, 37.1%), SDHC (N = 7, 10.0%), SDHA (N = 2, 2.9%). Additionally, three patients had multiple mutations, with one having an SDHA, TMEM127, and HOXB13 mutation, one with an SDHB and CHEK2 mutation, and one with an NF1 and BRCA2 mutation. Genetic germline mutations are common among patients with HNPGL, especially with SDHx mutations. Among the 119 patients who underwent genetic testing in our cohort, the rate of SDHx mutations was 83.0% in the group with multiple PGLs compared to 36.1% in the group with single PGL. In addition, SDHB and SDHD were by far the most commonly identified mutations in our cohort, including 30 (25.2%) patients with a SDHB mutation and 26 (21.8%) patients with a SDHD mutation. Compared to patients with SDHB mutations, patients with SDHD mutations had much higher odds of multiple tumors (OR 6.29, 95%CI 1.74–22.71, *p* < 0.01). SDHD mutations have been shown to be associated with more multifocality than SDHB mutations (74% vs. 28%, *p* < 0.01) [26,27]. In our cohort, patients who tested positive for genetic mutations were diagnosed at a significantly younger age compared to patients who tested negative for genetic mutation, 34.6 ± 13.7 vs. 57.6 ± 13.8 years (*p* < 0.01), respectively. Previously reported data from University of Pennsylvania by Fishbein et al. in 2013 comparing patients with germline mutations to those without an identified mutation revealed a similarly significant difference in age at diagnosis (30.44 ± 13.37 vs. 45.35 ± 14.99 years, *p* < 0.01) [28].

Among our cohort, 49 (26.2%) patients had multiple PGLs, including 18 patients with metachronous, 17 patients with synchronous, and 14 patients with both metachronous and synchronous presentation of PGLs. The rate of multiple PGLs in our cohort falls within the reported range of 16.5–58% by other larger retrospective studies [1,19,20,22]. Compared to patients with single PGL, patients with multiple PGLs had higher odds of having a genetic mutation (OR 6.45, 95%CI 2.64–15.73, *p* < 0.01). About 83.0% (39/47) of patients with multiple PGLs in our cohort tested positive for a mutation and the vast majority of these mutations were SDHD (22/39, 56.4%) and SDHB (14/39, 35.9%). Similarly, Künzel et al., in 2014, reported a series of 10 patients with multifocal HNPGLs and found a positive mutation rate of 4/6 in patients with completed testing, with the majority being SDHD (75%) mutations vs. SDHB mutations (25%) [21]. Papaspyrou et al., in 2012, reported a multifocal tumor rate of 18.9%, which was much higher when restricted to cases with SDHx-mutation positivity [19].

HNPGLs are mostly metabolically inactive. In our cohort, dopamine was the most commonly elevated plasma test. Metanephrines were rarely elevated in HNPGLs, with the exception of other HNPGLs. Compared to HNPGLs, non-HNPGL were more likely to have a plasma test of >3 ULN, especially for normetanephrine (38.5%) and dopamine (42.3%). Urine test was performed less frequently than plasma test (84 vs. 154 patients), but it had similar results of higher overall rates of elevated urine test (>3 ULN) in other HNPGL (27.3%) and non-HNPGL (37.5%). The overall rates of elevated urine tests (>3 UNL) in our cohort of CBP, JP, TP and VP were much lower, correlating with the low rates of biochemical activity among HNPGLs. Erickson et al. found that 34% of the patients screened preoperatively (N = 40/128) with a 24 h urine collection had significant elevations in either metanephrine, norepinephrine, epinephrine, or dopamine [1]. However, only 9 of the 40 had a HNPGL [1]. Smith et al. in 2021 reported on 152 patients with 182 HNPGLs that underwent urine or plasma biochemical test and reported a rate of 20.4% with elevated metabolite levels [29]. However, only 9.2% of patients experienced a clinically significant elevation resulting in symptoms [29].

Anatomic imaging in the form of CT and MRI was the most common diagnostic modality. MRI with gadolinium contrast is recommended for all HNPGLs with an emphasis on CT imaging of the temporal bone for improved visualization of TP or JP or for adrenal PGL [30,31]. The British Skull Base Society further recommends whole-body imaging for all tumors in the form of MRI or DOTATATE PET/CT [30]. Contrarily, data from Contrera et al. in 2019 evaluating 234 adults with a HNPGL showed an incidence of a secondary HNPGL to be relatively low at 1.7% at 5 years and 5.1% at 10 years, questioning the necessity of whole-body imaging for evaluation of multifocal disease in patients without familial disease [32]. The most commonly performed functional imaging study in our cohort was a DOTATATE/Octreoscan. Myssiorek et al. reviewed the literature on the use of nuclear medicine in evaluating HNPGLs and recommended using it in the setting of familial, SDHB mutation, malignant, or multifocal disease [33]. In our cohort, patients who underwent genetic screening (58/111, 52.3%) more frequently had an octreoscan than those without genetic screening (16/65, 24.6%). Additionally, patients with TPs least commonly underwent DOTATATE/Octreoscan (3/33, 18.2%), likely due to the low rates of genetic evaluation, as well as the low rates of multifocal or metastatic disease observed in TPs. DOTATATE has become more commonly used due to its high pooled detection rate, especially in comparison to MIBG [34]. Furthermore, DOTATATE has a much higher sensitivity than MIBG for PGLs, especially in the setting of SDHc-associated PGLs, which are more likely to be multifocal [34].

Surgery remains the most definitive and commonly used treatment for local disease control, especially in CBP or TP [30]. However, external beam radiation therapy (EBRT) and stereotactic radiosurgery (SRS) were more commonly used for tumors with a high risk of morbidity with surgery such as JP [30]. Systemic treatment was rarely used in our cohort. Almost all patients underwent local treatment (175/178, 98%) with either surgery or radiation therapy. Notably, JPs and VPs had an almost equal distribution between surgery and radiation therapy due to the risk for lower cranial neuropathies with surgical excision. Wanna et al., in 2014, proposed subtotal resection for large JPs with functional lower cranial nerves as a strategy to preserve cranial nerve function while significantly reducing tumor burden [35]. They found that an 80% reduction in tumor size was associated with no tumor growth over a 44.6-month period, and only two patients required radiotherapy [35]. To avoid initial radiation therapy, which only potentially stops the growth of these tumors and has long-term side effects, this strategy could be employed with careful discussion with the patient given the generally benign and slow-growing nature of PGLs. While our cohort had a low rate of radiation therapy as curative therapy, Mendenhall et al., in 2019, reported a series of 149 patients with 176 PGLs treated with radiation therapy with excellent local control (99%, 96%, 95%), distant metastasis-free survival (99%, 99%, 99%), and cause-specific survival (98%, 98%, 98%) over a 5-, 10-, and 15-year period [36]. Additionally, Lassen-Ramshad et al. have reported a series of 81 patients with 82 HNPGLs treated with EBRT or SRS radiation therapy [37]. In their longest follow-up period of >20 years, all four patients had disease progression, which potentially calls into question the durability of radiation therapy [37].

At our institution, all patients with HNPGLs are referred for genetic and biochemical evaluation. Additionally, a multidisciplinary tumor board convenes weekly to discuss the care of each patient to optimize outcomes.

Retrospective chart review studies inherently face limitations due to the inability to capture data that were unavailable at the time of the initial encounter, and especially with the rapid advancement and increased accessibility of genetic testing and ongoing identification of new mutations over the course of the past two decades. The earliest date of genetic evaluation in this study was 2002, which means that most patients with tumors discovered earlier than that date did not undergo genetic testing for evaluation of mutations. As such, the actual percentage of patients with genetic mutations in this cohort may be underestimated. Additionally, the present study includes patients who were evaluated at our institution, but some might have had partial care at outside institutions without full records available for review. Furthermore, due to the multiple decades over which the patients were evaluated at our institution and the changes in health records systems, there will inevitably be incomplete data on metabolic testing and imaging studies that were unavailable or technically less detailed, or treatment data that were unavailable. Despite these limitations, this study provides a large cohort to the growing literature within this field alongside extensive data in genetic analysis, metabolic testing, imaging, and treatment modalities.

## 6. Conclusions

Our experience highlights the needs for routine genetic and biochemical evaluation of patients with HNPGLs alongside a multidisciplinary team approach. Additionally, this comprehensive evaluation may lead to a more personalized evaluation and workup of a patient with HNPGL.

## Figures and Tables

**Table 1:** Characteristics of the study cohort per patient.

	All Patients (N = 187)	Genetic Testing (Yes) (N = 119)	Genetic Testing (No) (N = 68)	*p* Value	Mutation Yes (N = 70)	Mutation No (N = 49)	*p* Value
**Gender (N, %)**				<0.05			<0.01
Female	129 (69.0%)	76 (58.9%)	53 (41.1%)		37 (48.7%)	39 (51.3%)	
Male	58 (31.0%)	43 (74.1%)	15 (25.9%)		33 (76.7%)	10 (23.3%)	
**Race/Ethnicity (N, %)**				0.29			0.26
Caucasian	140 (74.9%)	93 (66.4%)	47 (33.6%)		58 (62.4%)	35 (37.6%)	
African American	38 (20.3%)	20 (52.6%)	18 (47.4%)		10 (50.0%)	10 (50.0%)	
Others	9 (4.8%)	6 (66.7%)	3 (33.3%)		2 (33.3%)	4 (66.7%)	
**Age of Initial Diagnosis (Years)**				<0.01			<0.01
Mean (SD)	48.8 (17.4)	44.1 (17.8)	57.2 (12.9)		34.6 (13.7)	57.6 (13.8)	
Range	10–82	10–82	30–82		10–79	24–82	
**Family History (N, %)**				<0.01			<0.01
Yes	33 (17.6%)	33 (100.0%)	0 (0.0%)		32 (97.0%)	1 (3.0%)	
No	154 (82.4%)	87 (56.5%)	67 (43.5%)		39 (44.8%)	48 (55.2%)	
**Number of PGLs per Patient (N)**				<0.01			<0.01
Mean (SD)	1.6 (1.4)	1.9 (1.6)	1.0 (0.2)		2.4 (1.9)	1.2 (0.6)	
Range	1–10	1–10	1–2		1–10	1–4	

**Table 2:** Clinical information by tumor type.

	Carotid Body (N = 108)	Jugular (N = 64)	Tympanic (N = 34)	Vagal (N = 23)	Other HNPGL (N = 17)	Non-HNPGL (N = 50)
**Age at Diagnosis (Years)**						
Mean (SD)	45.4 (15.9)	51.7 (17.7)	55.4 (13.7)	43.8 (18.5)	51.1 (11.2)	40.8 (15.5)
Range	15–81	11–82	10–76	15–79	31–77	11–69
**Gender (N, %)**						
Female	63 (58.3%)	43 (67.2%)	33 (97.1%)	13 (56.5%)	9 (50%)	27 (54.0%)
Male	45 (41.7%)	21 (32.8%)	1 (2.9%)	10 (43.5%)	8 (47%)	23 (46.0%)
**Race/Ethnicity (N, %)**						
Caucasian	82 (75.9%)	49 (76.6%)	23 (67.6%)	18 (78.3%)	11 (64.7%)	34 (68.0%)
African American	19 (17.6%)	14 (21.9%)	8 (23.5%)	4 (17.4%)	6 (35.3%)	16 (32.0%)
Others	7 (6.5%)	1 (1.5%)	3 (8.2%)	1 (4.3%)	0	0
**Family History (N, %)**						
Yes	30 (27.8%)	9 (14.1%)	1 (2.9%)	8 (34.8%)	7 (41.2%)	26 (52.0%)
No	78 (72.2%)	55 (85.9%)	33 (97.1%)	15 (65%)	10 (58.8%)	24 (48.0%)
**Genetic Testing (N, %)**						
Yes	87 (80.6%)	46 (71.9%)	8 (23.5%)	21 (91.3%)	15 (88.2%)	49 (98.0%)
No	21 (19.4%)	18 (28.1%)	26 (76.5%)	2 (8.7%)	2 (11.8%)	1 (2.0%)
**Mutation (N, %)**						
Yes	60 (69.0%)	27 (58.7%)	2 (25.0%)	17 (81.0%)	14 (93.3%)	44 (89.8%)
No	27 (31.0%)	19 (41.3%)	6 (75.0%)	4 (19.0%)	1 (6.7%)	5 (10.2%)

**Table 3:** Genetic status and tumor type.

	All PGLs (N = 296)	Carotid Body (N = 108)	Jugular (N = 64)	Tympanic (N = 34)	Vagal (N = 23)	Other HNPGL (N = 18)	Non-HNPGL (N = 50)
**Genetic Testing (N, %)**	227 (76.4%)	87 (80.6%)	46 (71.9%)	8 (23.5%)	21 (91.3%)	16 (88.9%)	49 (98.0%)
**SDHB**	55 (24.2%)	18 (20.7%)	14 (30.4%)	0	4 (19.0%)	7 (43.8%)	12 (24.5%)
**SDHD**	91 (40.1%)	38 (43.7%)	6 (13.0%)	2 (25.0%)	10 (47.6%)	5 (31.3%)	30 (61.2%)
**SDHC**	9 (4.0%)	0	5 (10.9%)	0	2 (9.5%)	1 (6.3%)	1 (2.0%)
**SDHA**	4 (1.8%)	1 (1.1%)	1 (2.2%)	0	1 (4.8%)	0	1 (2.0%)
**NF1**	1 (0.4%)	0	0	0	0	1 (6.3%)	0
**VHL**	1 (0.4%)	1 (1.1%)	0	0	0	0	0
**MEN1**	1 (0.4%)	0	1 (2.2%)	0	0	0	0
Others *	6 (2.7%)	5 (5.7%)	0	0	0	1 (5.9%)	0
**No Mutations (N, %)**	63 (27.8%)	27 (31.0%)	19 (41.3%)	6 (75.0%)	4 (19.0%)	2 (12.5%)	5 (10.2%)

* Others include HOXB13, CHEK2, BRCA2, TMEM127, BARD1, and MUTYH mutations.

**Table 4:** Genetic status and tumor presentation.

	Patients with Single PGL (N = 138)			Patients with Multiple PGLs (N = 49)					
	All (N = 138)	Metastatic (N = 10)	Recurrent (N = 12)	All (N = 49)	Metastatic (N = 9)	Recurrent (N = 9)	Synchronous Only (N = 17)	Metachronous Only (N = 18)	Synchronous & Metachronous (N = 14)
**Genetic Testing (N, %)**	72 (52.2%)	10 (100%)	6 (50.0%)	47 (95.9%)	9 (100%)	9 (100%)	17 (100%)	16 (88.9%)	14 (100%)
**SDHB**	16 (22.2%)	4 (40.0%)	2 (33.3%)	14 (29.8%)	3 (33.3%)	3 (33.3%)	6 (35.3%)	7 (43.8%)	1 (7.1%)
**SDHD**	4 (5.6%)	0	0	22 (44.9%)	5 (55.6%)	6 (66.7%)	6 (35.3%)	6 (37.5%)	10 (71.4%)
**SDHC**	5 (6.9%)	1 (10.0%)	1 (16.7%)	2 (4.3%)	0	0	1 (5.9%)	1 (6.3%)	0
**SDHA**	1 (1.4%)	0	0	1 (2.1%)	1 (11.1%)	0	0	0	1 (7.1%)
**NF1**	1 (1.4%)	1 (10.0%)	1 (16.7%)	0	0	0	0	0	0
**VHL**	1 (1.4%)	0	0	0	0	0	0	0	0
**MEN1**	1 (1.4%)	0	0	0	0	0	0	0	0
Others *	6 (8.3%)	1 (10.0%)	1 (16.7%)	0	0	0	0	0	0
**No mutations (N, %)**	41 (56.9%)	4 (40.0%)	2 (33.3%)	8 (17.0%)	0	0	4 (23.5%)	2 (12.5%)	2 (14.3%)

* Others include HOXB13, CHEK2, BRCA2, TMEM127, BARD1, and MUTYH mutations.

**Table 5:** Plasma biochemical profile by tumor type.

	All PGLs (N = 296)	Carotid Body (N = 108)	Jugular (N = 64)	Tympanic (N = 34)	Vagal (N = 23)	Other HNPGL (N = 17)	Non-HNPGL (N = 50)
**Metanephrine (N, %)**	142 (47.8%)	72 (66.7%)	52 (81.3%)	11 (32.4%)	19 (82.6%)	15 (88.2%)	26 (52.0%)
WNL	134 (94.4%)	68 (94.4%)	52 (100%)	10 (90.9%)	16 (84.2%)	12 (80.0%)	24 (92.3%)
1–3 ULN	7 (4.9%)	4 (5.6%)	0	1 (9.1%)	3 (15.8%)	2 (13.3%)	2 (7.7%)
>3 ULN	1 (0.7%)	0	0	0	0	1 (6.7%)	0
**Normetanephrine (N, %)**	142 (47.8%)	72 (66.7%)	52 (81.3%)	11 (32.4%)	19 (82.6%)	15 (88.2%)	26(52.0%)
WNL	89 (62.7%)	48 (66.7%)	30 (57.7%)	7 (63.6%)	10 (52.6%)	4 (26.7%)	8 (30.8%)
1–3 ULN	39 (27.5%)	17 (23.6%)	19 (36.5%)	3 (27.3%)	6 (31.6%)	6 (40.0%)	8 (30.8%)
>3 ULN	14 (9.9%)	7 (9.7%)	3 (5.8%)	1 (9.1%)	3 (15.8%)	5 (33.3%)	10 (38.5%)
**Epinephrine (N, %)**	144 (48.5%)	74 (68.5%)	52 (81.3%)	11 (32.4%)	19 (82.6%)	15 (88.2%)	26 (52.0%)
WNL	121 (84.0%)	64 (86.5%)	42 (80.8%)	10 (90.9%)	18 (94.7%)	9 (60.0%)	21 (80.8%)
1–3 ULN	20 (13.9%)	8 (10.8%)	9 (17.3%)	1 (9.1%)	1 (5.3%)	6 (40.0%)	4 (15.4%)
>3 ULN	3 (2.1%)	2 (2.7%)	1 (1.9%)	0	0	0	1 (3.8%)
**Norepinephrine (N, %)**	145 (48.8%)	75 (69.4%)	52 (81.3%)	11 (32.4%)	19 (82.6%)	15 (88.2%)	26 (52.0%)
WNL	82 (56.6%)	46 (61.3%)	26 (50.0%)	7 (63.6%)	12 (63.2%)	4 (26.7%)	11 (42.3%)
1–3 ULN	55 (37.9%)	24 (32.0%)	22 (42.3%)	4 (36.4%)	6 (31.6%)	8 (53.3%)	11 (42.3%)
>3 ULN	8 (5.5%)	5 (6.7%)	4 (7.7%)	0	1 (5.3%)	3 (20.0%)	4 (15.4%)
**Dopamine (N, %)**	146 (49.2%)	76 (70.4%)	52 (81.3%)	11 (32.4%)	19 (82.6%)	15 (88.2%)	26 (52.0%)
WNL	81 (55.5%)	44 (57.9%)	23 (44.2%)	8 (72.7%)	13 (68.4%)	5 (33.3%)	11 (42.3%)
1–3 ULN	27 (18.5%)	11 (14.5%)	10 (19.2%)	1 (9.1%)	3 (15.8%)	5 (33.3%)	4 (15.4%)
>3 ULN	38 (26.0%)	21 (27.6%)	19 (36.5%)	2 (18.2%)	3 (15.8%)	5 (33.3%)	11 (42.3%)
**Overall (N, %)**	154 (51.9%)	80 (74.1%)	55 (85.9%)	12 (35.3%)	19 (82.6%)	15 (88.2%)	26 (52.0%)
WNL	55 (35.7%)	33 (41.3%)	14 (25.5%)	6 (50.0%)	9 (47.4%)	2 (13.3%)	6 (23.1%)
1–3 ULN	50 (32.5%)	22 (27.5%)	19 (34.5%)	3 (25.0%)	6 (31.6%)	4 (26.7%)	3 (11.5%)
>3 ULN	49 (31.8%)	25 (31.3%)	22 (40.0%)	3(25.0%)	4 (21.1%)	9 (60.0%)	17 (65.4%)

**Table 6:** Urine biochemical profile by tumor type.

	All PGLs (N = 296)	Carotid Body (N = 108)	Jugular (N = 64)	Tympanic (N = 34)	Vagal (N = 23)	Other HNPGL (N = 17)	Non-HN PGL (N = 50)
**Metanephrine (N, %)**	70 (23.6%)	41 (38.0%)	22 (34.4%)	4 (11.8%)	8 (34.8%)	11 (61.1%)	16 (32.0%)
WNL	63 (90.0%)	37 (90.2%)	18 (81.8%)	4 (100%)	7 (87.5%)	10 (90.9%)	14 (87.5%)
1–3 ULN	6 (8.6%)	3 (7.3%)	4 (18.2%)	0	1 (12.5%)	1 (9.1%)	2 (12.5%)
>3 ULN	1 (1.4%)	1 (2.4%)	0	0	0	0	0
**Normetanephrine (N, %)**	70 (23.6%)	41 (38.0%)	22 (34.4%)	4 (11.8%)	8 (34.8%)	11 (61.1%)	16 (32.0%)
WNL	53 (75.7%)	30 (73.2%)	14 (63.6%)	3 (75.0%)	6 (75.0%)	6 (54.5%)	8 (50.0%)
1–3 ULN	13 (42.9%)	8 (19.5%)	5 (22.7%)	1 (25.0%)	2 (25.0%)	3 (27.3%)	5 (31.3%)
>3 ULN	4 (5.7%)	3 (7.3%)	3 (13.6%)	0	0	2 (18.2%)	3 (18.8%)
**Epinephrine (N, %)**	74 (24.9%)	41 (38.0%)	26 (40.6%)	3 (8.8%)	7 (30.4%)	11 (61.1%)	15 (30.0%)
WNL	69 (93.2%)	37 (90.2%)	24 (92.3%)	3 (100%)	7 (100%)	10 (90.9%)	12 (80.0%)
1–3 ULN	5 (6.8%)	4 (9.8%)	2 (7.7%)	0	0	1 (9.1%)	3 (20.0%)
>3 ULN	0	0	0	0	0	0	0
**Norepinephrine (N, %)**	74 (24.9%)	41 (38.0%)	27 (42.2%)	3 (8.8%)	7 (30.4%)	10 (55.6%)	15 (30.0%)
WNL	57 (77.0%)	28 (68.3%)	20 (74.1%)	3 (100%)	6 (85.7%)	5 (50.0%)	7 (46.7%)
1–3 ULN	13 (17.6%)	10 (24.4%)	5 (18.5%)	0	1 (14.3%)	4 (40.0%)	5 (33.3%)
>3 ULN	4 (5.4%)	3 (7.3%)	2 (7.4%)	0	0	1 (10.0%)	3 (20.0%)
**Dopamine (N, %)**	74 (24.9%)	41 (38.0%)	26 (40.6%)	3 (8.8%)	7 (30.4%)	11 (61.1%)	15 (30.0%)
WNL	54 (73.0%)	27 (65.9%)	20 (76.9%)	3 (100%)	5 (71.4%)	7 (63.6%)	8 (53.3%)
1–3 ULN	16 (21.6%)	12 (29.3%)	5 (19.2%)	0	2 (28.6%)	3 (27.3%)	4 (26.7%)
>3 ULN	4 (5.4%)	2 (4.9%)	1 (3.8%)	0	0	1 (9.1%)	3 (20.0%)
**Overall (N, %)**	84 (23.6%)	48 (44.4%)	29 (45.3%)	4 (11.8%)	8 (34.8%)	11 (61.1%)	16 (32.0%)
WNL	48 (57.1%)	25 (52.1%)	16 (55.2%)	3 (75.0%)	5 (62.5%)	5 (45.5%)	4 (25.0%)
1–3 ULN	27 (32.1%)	17 (35.4%)	9 (31.0%)	1 (25.0%)	3 (38.5%)	3 (27.3%)	6 (37.5%)
>3 ULN	9 (10.7%)	6 (12.5%)	4 (13.8%)	0	0	3 (27.3%)	6 (37.5%)

**Table 7:** (A) Diagnostic imaging utilization among the cohort. (B) Diagnostic imaging utilization by tumor type.

(A)							
	All Patients (N = 176)		Genetic Testing (No) (N = 65)	Genetic Testing (Yes) (N = 111)			
				Mutation Yes (N = 63)		Mutation No (N = 48)	
**Anatomic Imaging** **(N, %)**	**175 (99.4%)**		64 (98.5%)	63 (100%)		48 (100%)	
MRI (N, %)	117 (66.9%)		33 (51.6%)	49 (77.8%)		35 (72.9%)	
CT (N, %)	134 (76.6%)		49 (76.6%)	49 (77.8%)		36 (75.0%)	
Ultrasound (N, %)	26 (14.9%)		8 (12.5%)	8 (12.7%)		10 (20.8%)	
Angiogram (N, %)	29 (16.6%)		8 (12.5%)	14 (22.2%)		7 (14.6%)	
**Functional Imaging (N, %)**	86 (48.9%)		18 (27.7%)	40 (63.5%)		28 (58.3%)	
Octreoscan/DOTATATE (N, %)	74 (86.0%)		16 (88.9%)	31 (77.5%)		27 (96.4%)	
MIBG (N, %)	15 (17.4%)		2 (11.1%)	12 (30.0%)		1 (3.6%)	
FDG-PET (N, %)	7 (8.1%)		0	4 (10.0%)		3 (10.7%)	
FDPA-PET (N, %)	1 (1.2%)		0	0		1 (3.6%)	
**(B)**							
	**All PGLs** **(N = 258)**	**Carotid Body** **(N = 93)**	**Jugular** **(N = 58)**	**Tympanic** **(N = 33)**	**Vagal** **(N = 20)**	**Other HNPGL (N = 13)**	**Non-HN PGL (N = 40)**
**Anatomical Scan (N, %)**	237 (91.9%)	92 (98.9%)	57 (98.3%)	32 (97.0%)	19 (95.0%)	10 (71.4%)	27 (67.5%)
MRI	156 (65.8%)	51 (55.4%)	42 (73.7%)	16 (50.0%)	16 (84.2%)	9 (90.0%)	22 (81.5%)
CT	159 (67.1%)	63 (68.5%)	44 (77.2%)	25 (78.1%)	12 (63.2%)	7 (70.0%)	8 (29.6%)
Ultrasound	30 (12.7%)	30 (32.6%)	0	0	0	0	0
Angiogram	32 (13.5%)	21 (22.8%)	6 (10.5%)	1 (3.1%)	3 (15.8%)	0	1 (3.7%)
**Functional Scan (N, %)**	125 (48.4%)	39 (41.9%)	35 (60.3%)	6 (18.2%)	11 (55.0%)	6 (46.2%)	27 (67.5%)
Octreoscan/DOTATATE	96 (76.8%)	31 (79.5%)	32 (91.4%)	6 (100%)	8 (72.7%)	5 (83.3%)	13 (48.1%)
MIBG	26 (20.8%)	7 (17.9%)	2 (5.7%)	0	3 (27.3%)	2 (33.3%)	12 (44.4%)
FDG-PET	9 (7.2%)	2 (5.1%)	2 (5.7%)	0	1 (9.1%)	0	4 (14.8%)
FDPA-PET	1 (0.8%)	0	1 (2.9%)	0	0	0	0

**Table 8:** (A) Treatment modality among the cohort. (B) Treatment modality by tumor type.

(A)							
	All Patients (N = 178)		Genetic Testing (No) (N = 65)		Genetic Testing (Yes) (N = 113)		
					Mutation (Yes) (N = 70)		Mutation (No) (N = 43)
**Local Therapy (N, %)**	**175 (98.3%)**		65 (100%)		68 (97.1%)		42 (97.7%)
**Surgery**	145 (82.9%)		59 (90.8%)		62 (91.1%)		24 (57.1%)
Surgery alone	90 (62.1%)		44 (74.6%)		34 (54.8%)		12 (50.0%)
Surgery + Embolization	55 (37.9%)		15 (25.4%)		28 (45.2%)		12 (50.0%)
**Radiation**	40 (22.9%)		6 (9.2%)		16 (23.5%)		18 (42.9%)
EBRT	21 (52.5%)		1 (16.7%)		11 (68.8%)		9 (50.0%)
SRS	19 (47.5%)		5 (83.3%)		5 (31.3%)		9 (50.0%)
**Systemic Treatment (N, %)**	6 (3.4%)		0		4 (5.7%)		2 (4.7%)
**Radionuclide**	2 (33.3%)		0		2 (50.0%)		0
MIBG	2 (100%)		0		2 (100%)		0
**Medical Treatment**	4 (66.7%)		0		2 (50.0%)		2 (100%)
**(B)**							
	**All PGLs** **(N = 296)**	**Carotid Body** **(N = 108)**	**Jugular** **(N = 64)**	**Tympanic** **(N = 34)**	**Vagal (N = 23)**	**Other HNPGL (N = 17)**	**Non-HNPGL** **(N = 50)**
**Local Therapy (N, %)**	227 (76.7%)	83 (76.9%)	54 (84.4%)	33 (97.1%)	10 (43.5%)	12 (70.6%)	30 (60.0%)
**Surgery**	183 (80.6%)	73 (88.0%)	31 (57.4%)	32 (97.0%)	5 (50.0%)	10 (83.3%)	30 (100%)
Surgery alone	123 (67.2%)	33 (45.2%)	17 (54.8%)	32 (100%)	2 (40.0%)	9 (90.0%)	30 (100%)
Surgery + Embolization	58 (31.7%)	40 (54.8%)	14 (45.2%)	0	3 (60.0%)	1 (10.0%)	0
**Radiation**	44 (19.4%)	10 (12.0%)	23 (42.6%)	1 (3.1%)	5 (50.0%)	2 (16.7%)	0
EBRT	22 (50.0%)	8 (80.0%)	9 (39.1%)	0	4 (80.0%)	1 (50.0%)	0
SRS	19 (43.2%)	2 (20.0%)	14 (60.9%)	1 (100%)	1 (20.0%)	1 (50.0%)	0
**Systemic Therapy (N, %)**	8 (2.7%)	2 (2.4%)	2 (3.6%)	0	2 (16.7%)	2 (14.3%)	0
**Radionuclide**	3 (37.5%)	0	0	0	1 (50.0%)	2 (100%)	0
MIBG	3 (100%)	0	0	0	1 (100%)	2 (100%)	0
**Medical Treatment**	5 (62.5%)	2 (100%)	2 (100%)	0	1 (50%)	0	0
**Active Surveillance (N, %)**	60 (20.3%)	22 (20.4)	8 (12.5%)	1 (2.9%)	8 (34.8%)	3 (17.6%)	18 (36.0%)

## Data Availability

The datasets presented in this article are not readily available because the data are part of an ongoing study. Requests to access the datasets should be directed to the corresponding authors.

## References

[1] Erickson D., Kudva Y.C., Ebersold M.J., Thompson G.B., Grant C.S., van Heerden J.A., Young W.F. (2001). Benign paragangliomas: Clinical presentation and treatment outcomes in 236 patients. J. Clin. Endocrinol. Metab..

[2] Taieb D., Kaliski A., Boedeker C.C., Martucci V., Fojo T., Adler J.R., Pacak K. (2014). Current approaches and recent developments in the management of head and neck paragangliomas. Endocr. Rev..

[3] Kumar V., Kumar V., Abbas A.K., Aster J.C., Cotran R.S., Robbins S.L. (2021). Robbins and Cotran Pathologic Basis of Disease.

[4] Baysal B.E. (2002). Hereditary paraganglioma targets diverse paraganglia. J. Med. Genet..

[5] Fishbein L., Nathanson K.L. (2012). Pheochromocytoma and paraganglioma: Understanding the complexities of the genetic background. Cancer Genet..

[6] Sandow L., Thawani R., Kim M.S., Heinrich M.C. (2023). Paraganglioma of the Head and Neck: A Review. Endocr. Pract..

[7] Suarez C., Sevilla M.A., Llorente J.L. (2007). Temporal paragangliomas. Eur. Arch. Otorhinolaryngol..

[8] Sweeney A.D., Carlson M.L., Wanna G.B., Bennett M.L. (2015). Glomus tympanicum tumors. Otolaryngol. Clin. N. Am..

[9] Wanna G.B., Sweeney A.D., Haynes D.S., Carlson M.L. (2015). Contemporary management of jugular paragangliomas. Otolaryngol. Clin. N. Am..

[10] Galan S.R., Kann P.H. (2013). Genetics and molecular pathogenesis of pheochromocytoma and paraganglioma. Clin. Endocrinol..

[11] Carlson M.L., Sweeney A.D., Pelosi S., Wanna G.B., Glasscock M.E., Haynes D.S. (2015). Glomus tympanicum: A review of 115 cases over 4 decades. Otolaryngol. Head Neck Surg..

[12] Boedeker C.C., Hensen E.F., Neumann H.P., Maier W., van Nederveen F.H., Suarez C., Kunst H.P., Rodrigo J.P., Takes R.P., Pellitteri P.K. (2014). Genetics of hereditary head and neck paragangliomas. Head Neck.

[13] Dahia P.L. (2014). Pheochromocytoma and paraganglioma pathogenesis: Learning from genetic heterogeneity. Nat. Rev. Cancer.

[14] Castro-Vega L.J., Letouze E., Burnichon N., Buffet A., Disderot P.H., Khalifa E., Loriot C., Elarouci N., Morin A., Menara M. (2015). Multi-omics analysis defines core genomic alterations in pheochromocytomas and paragangliomas. Nat. Commun..

[15] Fishbein L. (2016). Pheochromocytoma and Paraganglioma: Genetics, Diagnosis, and Treatment. Hematol. Oncol. Clin. N. Am..

[16] Brito J.P., Asi N., Bancos I., Gionfriddo M.R., Zeballos-Palacios C.L., Leppin A.L., Undavalli C., Wang Z., Domecq J.P., Prustsky G. (2015). Testing for germline mutations in sporadic pheochromocytoma/paraganglioma: A systematic review. Clin. Endocrinol..

[17] McCaffrey T.V., Myssiorek D., Marrinan M. (2001). Head and neck paragangliomas: Physiology and biochemistry. Otolaryngol. Clin. N. Am..

[18] Baysal B.E. (2008). Clinical and molecular progress in hereditary paraganglioma. J. Med. Genet..

[19] Papaspyrou K., Mewes T., Rossmann H., Fottner C., Schneider-Raetzke B., Bartsch O., Schreckenberger M., Lackner K.J., Amedee R.G., Mann W.J. (2012). Head and neck paragangliomas: Report of 175 patients (1989–2010). Head Neck.

[20] Rijken J.A., de Vos B., van Hest L.P., Dreijerink K.M.A., den Heijer M., Wisselink W., Blom G.J., Hensen E.F., Leemans C.R. (2019). Evolving management strategies in head and neck paragangliomas: A single-centre experience with 147 patients over a 60-year period. Clin. Otolaryngol..

[21] Kunzel J., de Tristan J., Mantsopoulos K., Koch M., Baussmerth M., Zenk J., Iro H. (2014). Experiences in the treatment of patients with multiple head and neck paragangliomas. Am. J. Otolaryngol..

[22] Langerman A., Athavale S.M., Rangarajan S.V., Sinard R.J., Netterville J.L. (2012). Natural history of cervical paragangliomas: Outcomes of observation of 43 patients. Arch. Otolaryngol. Head Neck Surg..

[23] Smith J.D., Harvey R.N., Darr O.A., Prince M.E., Bradford C.R., Wolf G.T., Else T., Basura G.J. (2017). Head and neck paragangliomas: A two-decade institutional experience and algorithm for management. Laryngosc. Investig. Otolaryngol..

[24] Papaspyrou K., Mann W.J., Amedee R.G. (2009). Management of head and neck paragangliomas: Review of 120 patients. Head Neck.

[25] Lenders J.W.M., Kerstens M.N., Amar L., Prejbisz A., Robledo M., Taieb D., Pacak K., Crona J., Zelinka T., Mannelli M. (2020). Genetics, diagnosis, management and future directions of research of phaeochromocytoma and paraganglioma: A position statement and consensus of the Working Group on Endocrine Hypertension of the European Society of Hypertension. J. Hypertens..

[26] Neumann H.P., Pawlu C., Peczkowska M., Bausch B., McWhinney S.R., Muresan M., Buchta M., Franke G., Klisch J., Bley T.A. (2004). Distinct clinical features of paraganglioma syndromes associated with SDHB and SDHD gene mutations. JAMA.

[27] Williams M.D., Tischler A.S. (2017). Update from the 4th Edition of the World Health Organization Classification of Head and Neck Tumours: Paragangliomas. Head Neck Pathol..

[28] Fishbein L., Merrill S., Fraker D.L., Cohen D.L., Nathanson K.L. (2013). Inherited mutations in pheochromocytoma and paraganglioma: Why all patients should be offered genetic testing. Ann. Surg. Oncol..

[29] Smith J.D., Ellsperman S.E., Basura G.J., Else T. (2021). Re-evaluating the prevalence and factors characteristic of catecholamine secreting head and neck paragangliomas. Endocrinol. Diabetes Metab..

[30] Lloyd S., Obholzer R., Tysome J., Group B.C. (2020). British Skull Base Society Clinical Consensus Document on Management of Head and Neck Paragangliomas. Otolaryngol. Head Neck Surg..

[31] Lenders J.W.M., Eisenhofer G. (2017). Update on Modern Management of Pheochromocytoma and Paraganglioma. Endocrinol. Metab..

[32] Contrera K.J., Yong V., Reddy C.A., Berber E., Lorenz R.R. (2019). Second primary tumors in patients with a head and neck paraganglioma. Head Neck.

[33] Myssiorek D., Scharf S.C. (2023). Is Nuclear Imaging Important in the Management of Head and Neck Paragangliomas?. Laryngoscope.

[34] Taieb D., Hicks R.J., Hindie E., Guillet B.A., Avram A., Ghedini P., Timmers H.J., Scott A.T., Elojeimy S., Rubello D. (2019). European Association of Nuclear Medicine Practice Guideline/Society of Nuclear Medicine and Molecular Imaging Procedure Standard 2019 for radionuclide imaging of phaeochromocytoma and paraganglioma. Eur. J. Nucl. Med. Mol. Imaging.

[35] Wanna G.B., Sweeney A.D., Carlson M.L., Latuska R.F., Rivas A., Bennett M.L., Netterville J.L., Haynes D.S. (2014). Subtotal resection for management of large jugular paragangliomas with functional lower cranial nerves. Otolaryngol. Head Neck Surg..

[36] Mendenhall W.M., Morris C.G., Amdur R.J., Hitchcock K.E., Silver N.L., Dziegielewski P.T. (2019). Radiotherapy for benign head and neck paragangliomas. Head Neck.

[37] Lassen-Ramshad Y., Ozyar E., Alanyali S., Poortmans P., van Houtte P., Sohawon S., Esassolak M., Krengli M., Villa S., Miller R. (2019). Paraganglioma of the head and neck region, treated with radiation therapy, a Rare Cancer Network study. Head Neck.

